# The Impact of Integrating 3D-Printed Phantom Heads of Newborns with Cleft Lip and Palate into an Undergraduate Orthodontic Curriculum: A Comparison of Learning Outcomes and Student Perception

**DOI:** 10.3390/dj13070323

**Published:** 2025-07-16

**Authors:** Sarah Bühling, Jakob Stuhlfelder, Hedi Xandt, Sara Eslami, Lukas Benedikt Seifert, Robert Sader, Stefan Kopp, Nicolas Plein, Babak Sayahpour

**Affiliations:** 1 Department of Orthodontics, Johann-Wolfgang Goethe University, 60596 Frankfurt, Germany; 2 Studio Hedi Xandt, Graumannsweg 19, 22087 Hamburg, Germany; 3 Clinic for Oral, Maxillofacial, and Facial Surgery, University Hospital Basel, 4031 Basel, Switzerland; 4 Clinic for Maxillofacial and Plastic Surgery, Johann-Wolfgang Goethe University, 60629 Frankfurt, Germany

**Keywords:** cleft lip and palate, 3D printing, 3D rapid prototyping, prospective study, dental education

## Abstract

**Background/Objectives**: This prospective intervention study examined the learning effect of using 3D-printed phantom heads with cleft lip and palate (CLP) and upper jaw models with CLP and maxillary plates during a lecture for dental students in their fourth year at J. W. Goethe Frankfurt University. The primary aim was to evaluate the impact of 3D-printed models on students’ satisfaction levels along with their understanding and knowledge in dental education. **Methods**: Six life-sized phantom heads with removable mandibles (three with unilateral and three with bilateral CLP) were designed using ZBrush software (Pixologic Inc., Los Angeles, CA, USA) based on MRI images and printed with an Asiga Pro 4K 3D printer (Asiga, Sydney, Australia). Two groups of students (n = 81) participated in this study: the control (CTR) group (n = 39) attended a standard lecture on cleft lip and palate, while the intervention (INT) group (n = 42) participated in a hands-on seminar with the same theoretical content, supplemented by 3D-printed models. Before and after the session, students completed self-assessment questionnaires and a multiple-choice test to evaluate knowledge improvement. Data analysis was conducted using the chi-square test for individual questions and the Wilcoxon rank test for knowledge gain, with the significance level set at 0.05. **Results**: The study demonstrated a significant knowledge increase in both groups following the lecture (*p* < 0.001). Similarly, there were significant differences in students’ self-assessments before and after the session (*p* < 0.001). The knowledge gain in the INT group regarding the anatomical features of unilateral cleft lip and palate was significantly higher compared to that in the CTR group (*p* < 0.05). **Conclusions**: The results of this study demonstrate the measurable added value of using 3D-printed models in dental education, particularly in enhancing students’ understanding of the anatomy of cleft lip and palate.

## 1. Introduction

The teaching of orthodontics and related dental topics has evolved significantly with technological advancements. Traditional chalk-and-board methods have been largely replaced by modern presentation tools, commonly utilized in disciplines like anatomy, pathology, and embryology [1]. However, dental students often face challenges in understanding spatial relationships when relying solely on two-dimensional (2D) images for studying anatomy. This has prompted the exploration of three-dimensional (3D) learning options, such as 3D-printed models, augmented reality (AR), and virtual reality (VR), to enhance teaching methodologies and the student learning experience [2].

Since the introduction of additive manufacturing by pioneers like Masters, Hull, and Andre in the 1980s, 3D printing has gained remarkable popularity across various sectors, including aerospace, architecture, automotives, and education. This advanced technology allows digital imaging data, such as computed tomography (CT) or magnetic resonance imaging (MRI) scans, to be transformed into tangible, physical objects [3]. Today, numerous 3D printing technologies and open-source software solutions enable the cost-effective production of precise anatomical models, finding applications in surgical planning, patient education, and academic training. The recent literature also highlights a growing body of evidence supporting immersive technologies such as 3D printing, augmented reality (AR), and virtual reality (VR) in medical and dental education [4].

Three-dimensionally printed anatomical models have become increasingly popular for education purposes. These models not only simulate dental procedures but also provide students with multi-directional views, thereby improving their understanding of complex anatomical structures compared to traditional textbook-based 2D images [5,6,7]. Moreover, hands-on projects involving such models have been shown to foster critical thinking and teamwork among students [8]. By allowing tactile interaction, these models offer more accurate spatial visualization, enabling students to examine anatomical structures or pathologies from various perspectives [9]. This tactile experience enhances the comprehension of 3D anatomy, a critical skill for aspiring dental professionals [10].

In dental education, most of the practical content is taught through hands-on teachings, specifically by carrying out partner exercises, such as performing dental examinations on each other. However, teaching cleft lip and palate (CLP) presents a unique challenge: newborns with CLP are too delicate for undergraduate involvement in their treatment. As a result, CLP education is typically limited to seminars with a heavy theoretical focus and minimal practical experience. Consequently, students often struggle to grasp the complex anatomy and treatment of CLP patients. To address this gap, 3D-printed phantom heads and models can provide students with valuable hands-on experience, significantly enhancing their understanding and learning outcomes [10].

Therefore, the present study aimed to compare two methods of teaching, with and without 3D-printed models, on students’ understanding of this subject.

## 2. Materials and Methods

### 2.1. Study Design and Ethics

This monocentric prospective intervention study aimed to determine whether integrating 3D-printed models into dental education enhances students’ understanding of cleft lip and palate compared to traditional teaching methods. The study was approved by the ethics committee of the medical department of J. W. Goethe University Frankfurt am Main (Nr. 2023-1541; approval date: 16 November 2023). Informed consent was obtained from all students.

### 2.2. Study Groups

Two randomly assigned groups of students (n = 81) participated in this study: the control (CTR) group (n = 39) attended a standard lecture on cleft lip and palate based on a PowerPoint presentation, while the intervention (INT) group (n = 42) participated in a hands-on seminar with the same theoretical content, supplemented by a demonstration and hands-on exercises using 3D-printed phantom heads featuring unilateral and bilateral CLP, along with corresponding models of the maxilla for each phantom head and the molding appliances made specifically for this model. Participation in the study was voluntary after providing written and informed consent, which could be revoked at any time.

An online randomization tool, Sealed Envelope™ (https://www.sealedenvelope.com/simple-randomiser/v1/lists (accessed on 22 November 2023)), with simple randomization with a 1:1 allocation ratio was applied. The randomization sequence was generated electronically prior to participant assignment. Each participant was then sequentially assigned to one of the two groups according to this pre-generated list. No physical sealed envelopes or manual assignment procedures were used; the allocation was conducted directly based on the online-generated sequence by the study coordinator, who was not involved in delivering the lectures or assessments.

### 2.3. Inclusion Criteria

The study included male and female dental students in their 4th education year at J. W. Goethe University Frankfurt am Main. These students participated in the study and had little or no prior knowledge or understanding of cleft lip and palate.

### 2.4. Exclusion Criteria

The study excluded students from disciplines other than dentistry who were not enrolled in the relevant semester, who were not willing to participate in the study, or who had extensive knowledge or understanding of cleft lip and palate (e.g., students conducting their dissertation on the topic of CLP or those who were concurrently studying medicine or had completed a medical degree).

### 2.5. Sample Size Calculation

The power calculation was performed in collaboration with the Institute for Biostatistics and Mathematical Modeling of the Faculty of Medicine at J. W. Goethe University Frankfurt am Main and was based on a study by Kiesel et al. [11]. A sample size of 74 participants, with 37 students in each group, included in the study was sufficient to detect an effect size of 0.7 for the primary outcome (student satisfaction) with at least 80% power when using a two-tailed, two-sample *t*-test at a 5% significance level. For computing the effect size a standard deviation of 1.06 and a mean group difference of 0.7 were assumed. This group difference is slightly higher than in the reference study but still within the corresponding confidence interval. The sample size was calculated using BiAS for Windows software (version 11).

### 2.6. Fabrication of 3D Models

#### 2.6.1. Phantom Head

**Figure 1:**
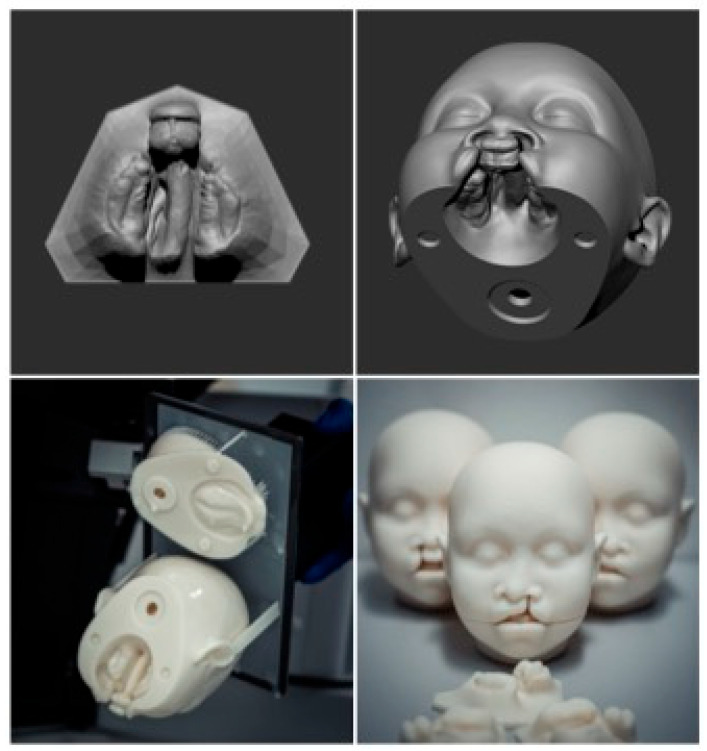
Clockwise: Socketed raw data of the 3D scan of bilateral CLP, a model view of the phantom head with an inserted intraoral scan, a view of the freshly printed model (upper and lower parts), and a view of the phantom head in three model variants: double-sided CLP, left-sided CLP, and right-sided CLP.

Six life-sized phantom heads, including three with unilateral and three with bilateral CLP, were designed using the CAD software ZBrush (Maxon Computer GmbH, Bad Homburg, Hesse, Germany (version 2023)) (Figure 1). The models were based on two MRI data sets (unilateral and bilateral CLP), providing highly detailed imaging of head structures. The design of the 3D phantom heads prioritized accurately replicating the characteristic extraoral morphology associated with cleft conditions. The phantom head was fitted with a type of joint mechanism using a plug-in connection system, enabling the detachable lower jaw to be easily removed (Figure 1).

Anonymized intraoral scans (IOSs) from standard diagnostic procedures for cleft-lip-and-palate patients of both cleft types, unilateral and bilateral, were incorporated in the corresponding digital 3D phantom head data sets (Figure 1). Removing the lower jaw provided an unobstructed view of the cleft upper jaw segments, accurately reflecting the specific cleft type as captured in the intraoral scan. Additionally, separate upper jaw models from the same IOS data sets were 3D-printed to enhance visualization.

The complete model was exported as an STL file and manufactured using a stereolithography (SLA) 3D printer (Asiga Pro 4K, Sydney, Australia). The printing process involved curing liquid resin layer-by-layer with UV light. After printing, the phantom head model was cleaned, refined, and smoothed to achieve a high-quality surface finish.

#### 2.6.2. Maxillary Plates

**Figure 2:**
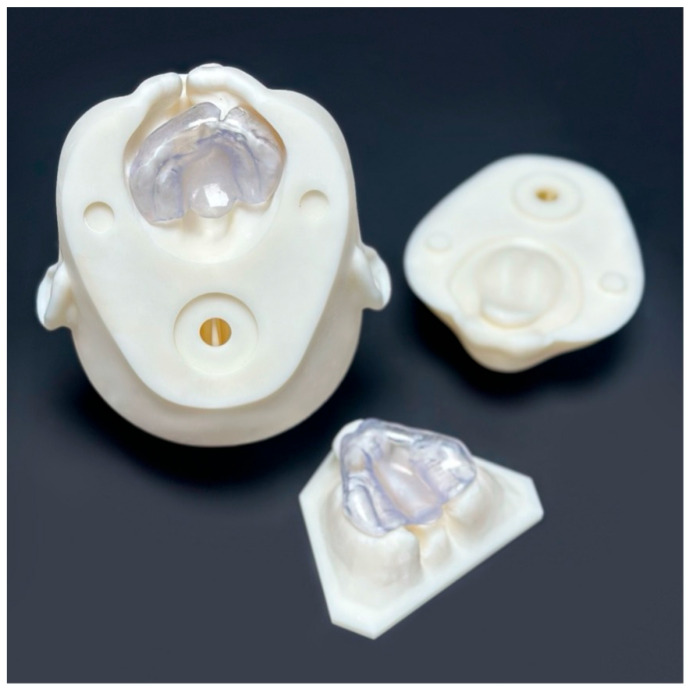
Top left: view of unilateral phantom head with plate inserted; top right: lower part of phantom head; bottom middle: 3D-printed model of double-sided CLP with maxillary plate.

Intraoral scans from standard diagnostic procedures for cleft-lip-and-palate patients with unilateral and bilateral cleft lip and palate were anonymized and used as the basis for the designing feeding plates. After preprocessing in the diagnostic software OnyxCeph^3TM^ (Image Instruments GmbH, Chemnitz, Germany; release version 3.2.223), digital dental models were printed. Passive plates were designed using the OnyxCeph^3TM^ software. The 3D design files were prepared for printing and uploaded to the printer software (Asiga©, Sydney, Australia; version 2.1.0). The passive plates were then printed using an Asiga Pro 4K 3D printer, cleaned with isopropanol, and cured. Final adjustments and polishing were performed in the dental laboratory. The active plates incorporated an active element, such as a screw, and were fabricated and polished in the in-house dental laboratory. All plates, regardless of their type, fit both the corresponding maxilla of the 3D-printed phantom heads and the separately printed maxillary models (Figure 2).

### 2.7. The Lecture Content

The lecture slides were the same in both seminars. The course presented an introduction to the topic of cleft lip and palate; accordingly, topics such as etiology, epidemiology, and manifestation were discussed and illustrated in a structured way in the presentation. A special focus was placed on teaching the anatomical features of cleft lip and palate and the corresponding PSIO therapeutic maxillary drinking plates. While this was only shown digitally as an illustration in the traditional seminar, the participants were able to hold and examine these 3D-printed heads in the seminar with integrated 3D foam models, take the two head shells apart, and inspect the individual anatomy of the split jaw segments. The maxillary drinking plates of the PSIO therapy could also be placed on and removed from the ridges of the printed foam models.

### 2.8. Data Collection

Two randomly assigned groups of students (n = 81) participated in this study. The control (CTR) group (n = 39) attended the standard lecture on cleft lip and palate based on a PowerPoint presentation, while the intervention (INT) group (n = 42) participated in a hands-on seminar with the same theoretical content, supplemented by a physical demonstration using 3D-printed phantom heads featuring unilateral and bilateral CLP, along with corresponding plates. Both groups received the same theoretical content with a duration of one hour and the same single instructor held both lectures.

The CTR and INT groups completed a course evaluation, a validated multiple-choice test (MCT), and a self-assessment questionnaire to measure satisfaction and knowledge improvement within the course (See Supplementary Materials).

**Figure 3:**
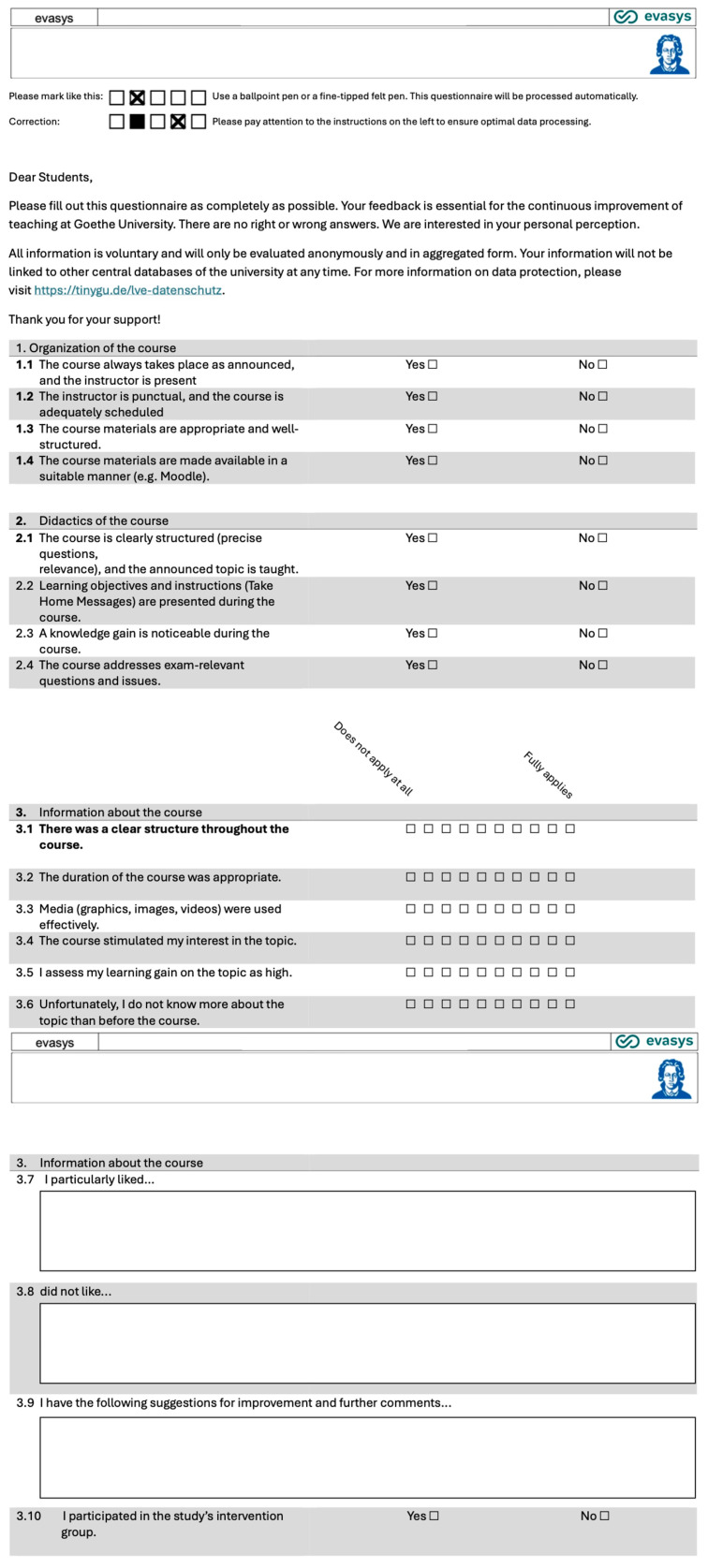
Standard evaluation form used for evaluation of seminar by both groups.

**Figure 4:**
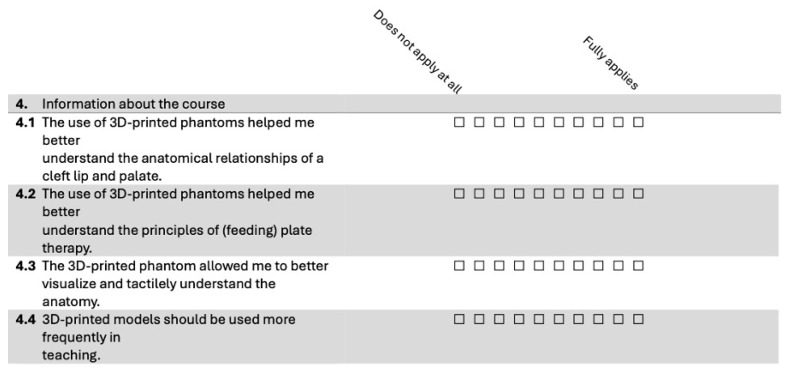
Additional evaluation questions for the intervention group.

**Course Evaluation:** All participants in both groups (CTR and INT) completed an evaluation form after the seminar, which included yes/no questions, 10-point Likert scale items ranging from “Does not apply at all” (1) to “Fully applies” (10), and free-text fields for qualitative feedback. While the free-text responses provided valuable qualitative insights, only the quantitative data were included in the descriptive statistical analysis. The evaluation form is shown in Figure 3, with additional questions for the INT group presented in Figure 4. The evaluation questionnaire was developed in collaboration with J. W. Goethe University Frankfurt am Main and was administered online.

Although inter-rater reliability was not applicable in this context—as only one instructor conducted all sessions and assessments—instructional consistency was further supported by using a script to guide the lecture content and interaction with the models. This helped minimize variation across sessions and ensured that all participants received the same educational experience within their assigned group.

To minimize assessment bias, blinding was implemented at the outcome assessment level. The lecturer who conducted both the control and intervention sessions did not participate in the evaluation of student performance. Instead, assessments were graded by independent evaluators who were completely blinded to the nature of the instructional sessions (i.e., whether 3D models were used), to the group assignments of the students, and to the experimental hypothesis.

All post-intervention assessments were anonymized prior to evaluation, and no identifying or group-related information was available to the assessors during the scoring process. This blinding strategy was designed to ensure impartial evaluation and to strengthen the internal validity of our findings.

**Knowledge Questionnaire (MCT):** A multiple-choice questionnaire (MCT) consisting of 14 questions was administered both before and after the seminar to assess students’ knowledge of CLP. The questions covered topics such as anatomy, feeding plates, and early interventions, allowing for a comparison of knowledge gains between the CTR and INT groups.

The questionnaire used in this study was adapted from the standardized internal question pool of the university’s dental faculty. This question pool is routinely used in summative assessments and is constructed in alignment with the national learning framework and educational objectives defined by the German Ministry of Health and the institutional academic board. Although the instrument has not undergone formal psychometric validation, its structure and content reflect the recognized standards of the university’s examination system.

Self-Assessment Questions: Students completed five self-assessment questions before and after the seminar (Section 3.3). These questions addressed their satisfaction with their knowledge of and interest in CLP, and their understanding of key concepts, including the types of CLP, the importance of multidisciplinary care, and the roles of professionals involved in CLP treatment. Responses were recorded on a five-point Likert scale ranging from “Strongly disagree” (1) to “Strongly agree” (5).

### 2.9. Statistical Analysis

The statistical evaluation was carried out under the supervision of the Institute for Biostatistics and Mathematical Modeling of the Faculty of Medicine at J. W. Goethe University Frankfurt am Main. All analyses were performed using Jamovi version 2.3.28.0. To determine the appropriate statistical methods, the Shapiro–Wilk test was first used to assess the normality of the data. For normally distributed differences, a paired *t*-test was employed to compare pre- and post-seminar scores within each group. When the normality assumption was violated, the Wilcoxon signed-rank test was used as a non-parametric alternative.

The analysis encompassed three key areas: (1) the evaluations of the course, (2) the 14 individual knowledge questions, and (3) the 5 self-assessment questions regarding knowledge of and interest in CLP. Comparisons between the CTR and INT groups for individual knowledge questions and self-assessment responses were conducted using the Wilcoxon-Mann–Whitney U test. Descriptive statistics, including the mean values (mv), medians (md), and standard deviations (sd), were calculated to summarize the data and assess variability.

The significance level for all statistical tests was set at *p* = 0.05. The analyses ensured rigorous handling of the data and were conducted in compliance with standard statistical practices.

## 3. Results

### 3.1. Students’ Satisfaction with the Training Seminar

The evaluation of course organization and didactics reveals strong comparability between the CTR and INT groups, as shown in Table 1 and Table 2. Both groups provided similarly high ratings across all aspects of the course, including punctuality, clarity of structure, relevance of materials, and overall organization. For example, the mean scores for statements such as “The course takes place as announced, and the instructor is present” and “The course is clearly structured, and the announced topic is taught” were nearly identical, with minimal variation in standard deviations between the two groups.

**Figure 5:**
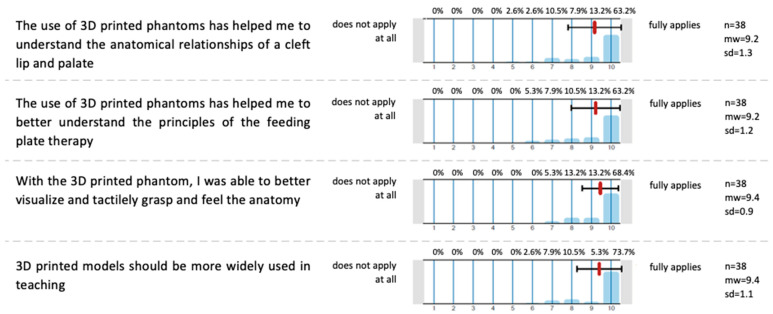
A self-assessment questionnaire with additional questions for the INT group to evaluate the use of 3D-printed phantoms. Answer possibility: 10-point Likert scale; 0 (Strongly disagree)–10 (Strongly agree); n = 38.

The INT group received additional questions about the use of the 3D phantoms, as displayed in Figure 5.

Across all four evaluation items, the median scores ranged from 9.2 to 9.4 on a 10-point Likert scale, with low standard deviations (0.9 to 1.3), indicating a strong consensus among participants. Students agreed that the phantom heads significantly enhanced their understanding of the anatomical relationships in cleft lip and palate (median = 9.2, SD = 1.3) and improved their comprehension of the principles of CLP plate therapy (median = 9.2, SD = 1.2). Additionally, they reported that the models helped them better visualize and tactilely grasp the anatomy (median = 9.4, SD = 0.9). Participants also strongly supported the broader integration of 3D-printed phantoms in teaching (median = 9.4, SD = 1.1). These results demonstrate the seminar’s success in effectively utilizing 3D-printed models to enhance student learning and satisfaction.

### 3.2. Increase in Knowledge

The secondary outcome, students’ knowledge of cleft lip and palate, was measured before and after the course using an MCT with 14 questions.

Table 3 presents the results of a paired Wilcoxon test conducted to evaluate the improvement in responses to individual knowledge questions before and after the seminar for both the CTR and INT groups. Each row corresponds to a specific question, with the Wilcoxon W statistic and *p*-value indicating whether the improvement was statistically significant. For both groups, all individual questions showed significant improvement, as reflected by *p*-values below 0.001. The “Total” row aggregates the results for all questions combined, showing no significant overall improvement for either group (*p* = 0.155 for CTR and *p* = 0.167 for INT). These results highlight that both the CTR and INT groups significantly improved their performance on each specific knowledge question after the seminar.

The following section shifts the focus from pre- vs. post-seminar comparisons to an analysis of differences between the control (CTR) and intervention (INT) groups for all 14 knowledge questions. Among these, only Questions 9 and 10 showed statistically significant differences, highlighting the educational impact of the 3D-printed phantoms used by the INT group. The other 12 questions revealed no significant differences between the groups.

**Figure 6:**
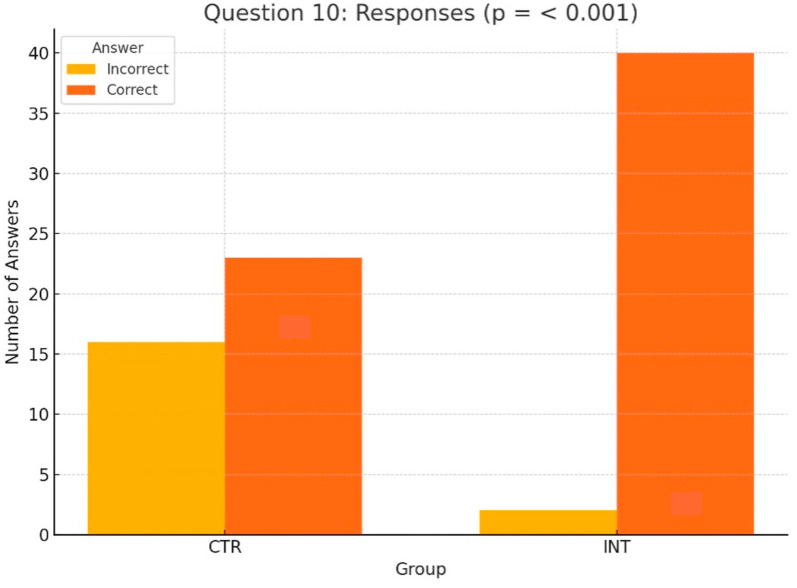
Distribution of correct and incorrect responses for Question 10 (‘anatomical peculiarities of a unilateral cleft lip’) in the CTR and INT groups (*p* < 0.001).

**Figure 7:**
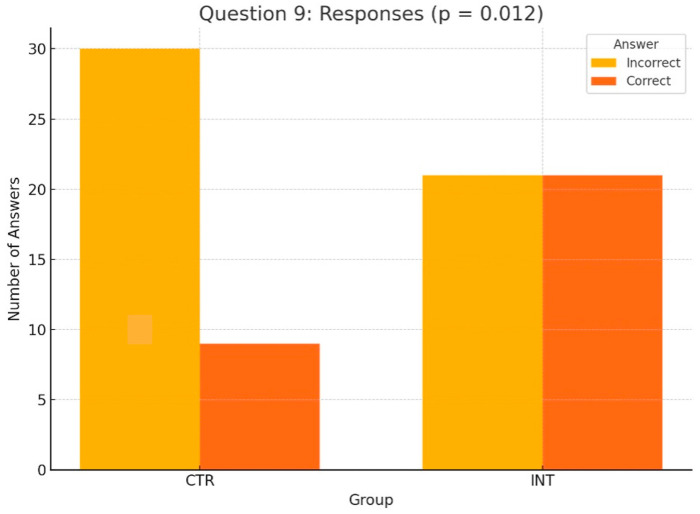
Distribution of correct and incorrect responses for Question 9 (‘use of feeding plates for cleft lip and palate’) in the CTR and INT groups (*p* = 0.012).

For Question 10 (Figure 6), which assessed students’ understanding of the anatomical peculiarities of a unilateral cleft lip and palate, the INT group significantly outperformed the CTR group, with 40 correct answers compared to 23 (*p* < 0.001, Wilcoxon test). Similarly, for Question 9 (Figure 7), which evaluated knowledge of feeding plates for cleft lip and palate, the INT group achieved 21 correct responses compared to 9 in the CTR group (*p* = 0.012, Wilcoxon test).

### 3.3. Improvement in Self-Assessment Scores Following the Seminar: CTR vs. INT Group

Students’ self-assessment of their knowledge of and interest in CLP was measured before and after the seminar using the questions outlined in Table 4. The analysis, summarized in Table 5, shows statistically significant improvements across all individual questions for both the CTR and INT groups, as well as for the combined total of all questions.

For the CTR group, the Wilcoxon W values ranged from 0.641 to 0.883 across individual questions, with *p*-values consistently below 0.001, demonstrating significant pre- to post-seminar gains in perceived knowledge and interest. The overall result for the combined questions was W = 0.748, *p* < 0.001, further supporting the effectiveness of the seminar for the CTR group.

Similarly, the INT group showed significant improvements across all questions, with Wilcoxon W values ranging from 0.522 to 0.915. The *p*-values were also significant, ranging from <0.001 to 0.013 for individual questions, and the combined result for all questions was W = 0.838, *p* < 0.001. Notably, the INT group demonstrated higher Wilcoxon W values on several individual questions compared to the CTR group, such as Question E (W = 0.915 for INT vs. W = 0.861 for CTR), reflecting stronger perceived improvements.

## 4. Discussion

Both groups gave equally high ratings for all aspects of the course, indicating consistent evaluations of course organization and didactics. This suggests that the observed differences in areas such as knowledge acquisition or understanding of specific topics can be attributed to the use of 3D-printed phantoms in the intervention group, rather than differences in the overall quality of course delivery. Students in the INT group reported that the 3D-printed phantoms enhanced their understanding of cleft anatomy and CLP plate therapy, providing improved visualization and tactile learning. These results support previous findings demonstrating that 3D-printed models can improve learner engagement and anatomical understanding in both dental and surgical training [6,7,10,12,13,14,15]. Student endorsement of the broader use of such models is consistent with outcomes from studies by Chen et al. and Chou et al., which emphasized the added value of hands-on simulation in cleft-related education and parental communication [13,16].

Student knowledge on CLP was assessed before and after the course using a 14-question MCT. Both groups showed significant improvement on all individual questions, though no significant overall improvement was observed for either group. Following the seminar, both the CTR and INT groups were able to significantly improve their results on each specific knowledge question after the seminar. These findings align with the work of Dalgalı et al. [17], who reported that 3D-model-assisted learning resulted in better short-term test scores, though long-term differences between educational methods were not significant. Our results further support the notion that while physical models enhance topic-specific understanding, they may not universally improve all knowledge domains without long-term reinforcement [10,13,17].

Focusing on the pre- vs. post-seminar comparisons between the CTR and INT groups, Questions 9 and 10 showed statistically significant differences, highlighting the educational impact of the 3D-printed phantoms used by the INT group. For Question 10, which focused on the anatomical characteristics of a unilateral cleft lip and palate, the INT group demonstrated significantly better performance than the CTR group (*p* < 0.001). Similarly, for Question 9, assessing knowledge of maxillary plates for cleft lip and palate, the INT group achieved 21 correct responses compared to 9 in the CTR group (*p* = 0.012). These findings emphasize the value of hands-on learning tools, particularly 3D phantom heads, in enhancing students’ understanding of complex anatomical features and presurgical feeding plate therapy. The other 12 of 14 questions revealed no significant differences between the groups. This is consistent with prior simulation-based studies showing that 3D-printed models improve specific cognitive domains such as spatial anatomy and procedural steps [10,13,14,15]. In particular, Teuber Lobos et al. and Wright et al. showed that the inclusion of cleft simulation models leads to notable improvements in anatomical recognition and surgical planning [14,15].

Students’ self-assessment of their knowledge of and interest in CLP was measured before and after the seminar and showed statistically significant improvements across all individual questions for both the CTR and INT groups, as well as for the combined total of all questions. While for both groups, significant pre- to post-seminar gains in perceived knowledge and interest could be demonstrated, the INT group demonstrated higher Wilcoxon W values on several individual questions compared to the CTR group, reflecting stronger perceived improvements. These findings emphasize the substantial impact of the seminar on students’ self-perceived knowledge and engagement with cleft lip and palate topics, as observed in both groups. These results also highlight the additional educational benefits experienced by the INT group, which used 3D-printed phantoms to enhance learning. This comprehensive analysis underscores the seminar’s effectiveness in meeting its educational objectives. This is in agreement with Chou et al. [16], who reported that tactile simulation models not only improved learner confidence but also enhanced communication with patients’ families. These findings suggest that the use of 3D-printed models contributes to stronger affective engagement and perceived competency, which are essential components of learner-centered clinical education [10,13,16].

Although the use of 3D models improved performance on selected questions—particularly those involving anatomical understanding—no significant difference was observed in overall knowledge scores between groups. This observation is in line with Dalgalı et al. [17], who found that although 3D-model-supported education led to significant short-term gains, long-term knowledge retention did not differ significantly between the intervention and control groups. These results highlight the importance of instructional design: foundational topics such as etiology and epidemiology may benefit more from lectures and visual aids, while 3D models are particularly effective in promoting spatial and structural understanding [1,6,10,12]. Importantly, students in the INT group reported higher engagement and satisfaction, a pattern also noted in prior studies using hands-on 3D tools in dental education [7,8,10].

In diverse fields like aerospace, architecture, and automotives, additive manufacturing has rapidly gained traction. This also applies to education. Advanced digital technology allows digital imaging data, including CT and MRI scans, to be transformed into tangible, physical models [3]. Today, a wide range of 3D printing technologies and open-source software tools offer the ability to produce cost-effective and highly accurate anatomical models, and are already applied in many areas of medical education. Models of the heart, the face, bone, eyes, arteries, the pelvis, the liver, the chest, and the skull are used for surgical planning, patient education, and academic training [4,12].

Three-dimensionally printed anatomical models are gaining popularity, especially in dental education, for their ability to enhance learning experiences. Unlike traditional 2D textbook images, these models offer multi-directional views, improving students’ understanding of complex anatomical structures [5,6,7]. Additionally, hands-on lectures using these models promote critical thinking and teamwork [8]. By enabling tactile interaction, they provide more accurate spatial visualization, allowing students to explore anatomical features or pathologies from various angles—an essential skill for future dental professionals [9,10]. Seifert et al. [6] conducted a scientific study comparing various learning objectives for undergraduate dental students, including mucoperiosteal flap dissection, osteotomy of impacted third molars, free-mucosal-graft dissection, and root tip resection. A comparison was made between 3D-printed, patient-specific models and traditional cadaveric models. While cadaveric models provided superior haptic feedback for soft tissues, the 3D-printed patient-specific models were rated significantly higher in terms of anatomical accuracy, range of motion, and the realism of the surgical simulation [6]. In a scientific study by Richter et al. [7], thirteen dentists and twenty-seven undergraduate dental students compared 3D-printed tooth models to a commercial model in a conservative dentistry course. The participants excavated carious lesions, performed a cavity preparation, and restored defects, and rated 3D-printed models as significantly more realistic in terms of tactility and the distinction of color. In comparison to commercial models, the 3D-printed models were exceptionally cost-efficient.

Considering cleft lip and palate, 3D-printed models are currently applied mainly in the training of surgical techniques in oral and maxillofacial surgery. Chen et al. [13] assessed the integration of a printed cleft lip silicone simulation model in personalized surgical training and showed that the experimental group exhibited higher confidence levels in cleft lip surgery and anatomical landmark identification compared to the control group receiving a lecture based on a PowerPoint presentation. Wright et al. [14] conducted a validation study with five junior and five senior plastic surgery residents on a craniofacial bony and soft tissue anatomical model for use in simulating the performance of a fronto-orbital advancement. The simulation and the demonstration significantly improved the average score of the knowledge assessment of the junior residents. In a study by Teuber Lobos et al. [15], two groups of trainee and expert surgeons found a cleft-lip-and-palate hybrid simulation model to be a useful and appropriate teaching tool in cleft-related anatomy and surgical techniques. Chou et al. [16] showed that 3D-printed models provide the parents of patients with cleft lip and palate with a better understanding of their child’s condition before surgical intervention.

The integration of 3D-printed phantom models in the teaching of cleft lip and palate is also gaining attention in orthodontic curricula. However, the current body of research in this regard is still limited. In accordance with our findings, an investigation by AlAli et al. [10] proved that the use of 3D-printed models in educational seminars on cleft lip and palate resulted in a significant improvement in gained knowledge. Similarly to our findings, AlAli et al. demonstrated that their intervention group achieved higher scores in post-seminar assessments compared to their control group. In contrast to our study, the participants in the intervention group showed a higher level of satisfaction than the students in the control group.

It must be acknowledged that our study represents a short-term assessment, as knowledge was tested on the same day before and after the learning session. A study by Dalgali et al. [17] from 2024 evaluated the impact of teaching with 3D-printed dental models of patients with cleft lip and palate and e-learning-supported education after training at three timepoints. The 3D-model-assisted learning group achieved significantly higher short-term test results compared to the e-learning group, while, contrary to our research, no significant differences were found between their scores and those of the baseline group after the training. Regarding long-term learning one week later and late long-term learning one month later, no significant differences between the education methods could be found. Further orthodontic education evaluation studies with long-term learning effects would be desirable for future studies, as 3D-printed phantom models of cleft lip and palate present a realistic and cost-efficient tool in the training of orthodontics.

This study could emphasize the value of hands-on learning tools, particularly 3D phantoms, in enhancing students’ understanding of complex anatomical features and presurgical maxillary plate therapy. Moreover, the evaluation of the INT group could show that the integration of these models into teaching highlights the seminar’s success in enhancing learning and satisfaction. Three-dimensional phantoms should be adopted in future orthodontic training, setting a new standard for teaching complex anatomical and pathological concepts such as those associated with cleft lip and palate.

### Limitations

Even though the same instructor conducted both the control and intervention sessions to ensure consistency, the rather small number of study participants and the limitation of the participants to only one university probably reduced the generalizability of our results.

While the questionnaire was based on the university’s standardized exam question pool and aligned with national learning objectives, it has not been validated through formal psychometric testing. This represents a limitation in terms of external generalizability and measurement precision. Nevertheless, its alignment with official educational standards supports its relevance for evaluating student performance within the given institutional context.

Furthermore, it must be acknowledged that our research only evaluated a short-term assessment.

Gained knowledge should be tested in the long term, repeating the questionnaire several times. Data were analyzed per protocol, which always carries a risk for bias.

## 5. Conclusions

While our findings suggest that the use of 3D-printed anatomical models can enhance student engagement and improve their understanding of anatomically complex content such as cleft lip and palate, we recommend interpreting these results with caution. The study was conducted at a single institution with a limited sample size, and curricular frameworks and teaching approaches may differ significantly across dental schools. As such, the applicability of these findings to other educational contexts remains to be further explored. Nonetheless, 3D models appear to offer a promising supplementary tool in dental education that may enrich conventional lecture formats and support deeper learning in targeted areas.

## Figures and Tables

**Table 1:** Evaluation of course organization by control (CTR) group and intervention (INT) group. mv = mean value; md = median; sd = standard deviation.

Organization of the Course	CTR (n = 33)					INT (n = 40)				
	yes	no	mv	md	sd	yes	no	mv	md	sd
The course takes place as announced, and the instructor is present			1.0	1.0	1.0			1.0	1.0	0.0
The instructor is punctual, and the course is adequately scheduled	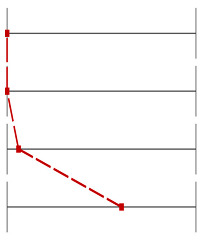		1.0	1.0	0.0	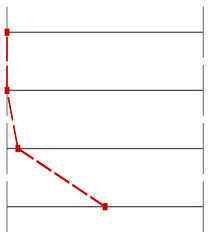		1.0	1.0	0.0
The course materials are appropriate and well-structured			1.1	1.0	0.2			1.1	1.0	0.2
The course materials are made available in a suitable manner (e.g., Moodle)			1.6	2.0	0.5			1.4	1.0	0.5

**Table 2:** Evaluation of course didactics by control (CTR) group and intervention (INT) group. mv = mean value; md = median; sd = standard deviation.

Didactics of the Course	CTR (n = 33)					INT (n = 40)				
	yes	no	mv	md	sd	yes	no	mv	md	sd
The course is clearly structured (precise questions, relevance), and the announced topic is taught.	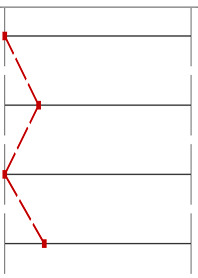		1.0	1.0	0.0			1.0	1.0	0.0
Learning objectives and instructions (Take Home Messages) are presented during the course.			1.2	1.0	0.4	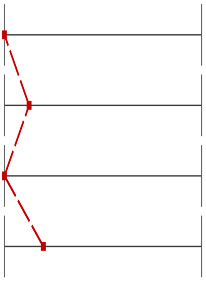		1.1	1.0	0.3
A knowledge gain is noticeable during the course.			1.0	1.0	0.0			1.0	1.0	0.0
The course addresses exam-relevant questions and issues.			1.2	1.0	0.4			1.2	1.0	0.4

**Table 3:** Paired Wilcoxon test results for pre- and post-seminar improvements in knowledge questions.

Group	Question (Pre- and Post-Seminar)	Wilcoxon W	*p* Value	Group	Question (Pre- and Post- Seminar)	Wilcoxon W	*p* Value
CTR	1	0.587	<0.001	INT	1	0.433	<0.001
	2	0.754	<0.001		2	0.738	<0.001
	3	0.789	<0.001		3	0.795	<0.001
	4	0.735	<0.001		4	0.739	<0.001
	5	0.693	<0.001		5	0.564	<0.001
	6	0.707	<0.001		6	0.757	<0.001
	7	0.611	<0.001		7	0.635	<0.001
	8	0.682	<0.001		8	0.497	<0.001
	9	0.797	<0.001		9	0.770	<0.001
	10	0.549	<0.001		10	0.683	<0.001
	11	0.698	<0.001		11	0.760	<0.001
	12	0.724	<0.001		12	0.740	<0.001
	13	0.677	<0.001		13	0.737	<0.001
	14	0.477	<0.001		14	0.502	<0.001
	Total	0.961	0.155		Total	0.959	0.167

**Table 4:** Self-assessment questions on knowledge of and interest in CLP.

Questions
(A) I am satisfied with my level of knowledge about cleft lip and palate.
(B) I am interested in the topic of cleft lip and palate.
(C) I can name the different types of cleft lip and palate and explain their differences.
(D) I can explain the importance of early intervention and multidisciplinary care in treating cleft lip and palate.
(E) I have basic knowledge of the roles of speech therapists, orthodontists, and other professionals in the cleft lip and palate care team.

*Answer Options:* Strongly agree (5), Agree (4), Neutral (3), Disagree (2), Strongly disagree (1).

**Table 5:** Pre- and post-seminar statistical results for self-assessment questions in CTR and INT groups.

Group	Question	W	*p*	Group	Question	W	*p*
CTR	A	0.883	<0.001	INT	A	0.810	<0.001
	B	0.641	<0.001		B	0.522	<0.001
	C	0.871	<0.001		C	0.874	0.001
	D	0.851	<0.001		D	0.905	0.007
	E	0.861	<0.001		E	0.915	0.013
	All questions	0.748	<0.001		All questions	0.838	<0.001

## Data Availability

The data is available upon request from the corresponding author.

## Supplementary Materials

The following supporting information can be downloaded at: https://www.mdpi.com/article/10.3390/dj13070323/s1, Anonymous Questionnaire on the Lecture about Cleft Lip and Palate.
