# Supplementary material for: The Impact of Integrating 3D-Printed Phantom Heads of Newborns with Cleft Lip and Palate into an Undergraduate Orthodontic Curriculum: A Comparison of Learning Outcomes and Student Perception
Source: Dent J (Basel). 2025 Jul 16;13(7):323. doi: 10.3390/dj13070323 (PMC12293818; doi:10.3390/dj13070323)
Supplement: Supplementary file 1 [file dentistry-13-00323-s001.zip › dentistry-3689293-supplementary.pdf]

Anonymous Questionnaire on the Lecture about Cleft Lip and Palate

Date: ________________

Semester:	Group:	Time of assessment:	Consent to participate in the study:
☐ 7<br>☐ 8	☐ Intervention<br>☐ Control	☐ Prior to Seminar<br>☐ After the Seminar	☐ Yes<br>☐ No
Age:	Gender:	Knowledge level on cleft lip and palate	Student or graduate of an additional major (for example medicine)
☐ < 21<br>☐ 21 - 24<br>☐ 24 - 28<br>☐ > 28	☐ male<br>☐ female<br>☐ divers	☐ high<br>☐ low	☐ Yes<br>☐ No<br>In case of Yes, which major:<br>________________________________

Hinweis: Setzen Sie ein Kreuz, wenn die Antwort zutrifft; Mehrfachnennungen sind möglich.

1. Which of the following is not a form of an incomplete cleft?

☐ Aleolar and Lip cleft
☐ Lip cleft
☑ Lip, alveolar and palate cleft
☐ palate cleft

2. What are the indications for primary treatment in patients with cleft lip and palate?

☑ Restoration of anatomical and functional structures of the orofacial region
☑ Support of hearing development
☐ Correction of primary crossbite
☑ Support of speech development

3. What are the functions of a feeding plate used in primary treatment?

☐ Enable a dorsal-caudal tongue position
☐ Control mandibular growth to harmonize form
☑ Facilitate feeding
☑ Separate oral and nasal cavities

4. What are the goals of surgery in cleft lip and palate patients?

- ☑ Closure of the lip
- ☐ Bridging the jaw cleft with hard tissue
- ☑ Reconstruction of the nasal floor
- ☐ Preservation of the lingual frenulum

5. What is the prevalence of cleft lip and palate?

- ☐ 1:3000 – 1:5000
- ☑ 1:500 – 1:700
- ☐ 1:800 – 1:900
- ☐ 1:50 – 1:70

6. What is the prevalence of isolated cleft palate?

- ☑ 1:1500
- ☐ 1:150
- ☐ 1:2500
- ☐ 1:250

7. How many hours per day should the feeding plate remain in the mouth?

- ☐ During the day (~12 hrs); should not be worn at night due to aspiration risk
- ☐ If possible, 16 hrs; remove during feeding
- ☑ Ideally 24 hrs; remove only for cleaning
- ☐ Only at night to avoid impairing speech development

8. How often should an active feeding plate be adjusted?

- ☐ 1–2 times daily open/close
- ☐ Once a week
- ☑ Every 2–3 days
- ☐ Once a month

9. The retention of the feeding plate in the patient’s mouth...

☑ ... depends on the precision of the intraoral scan
☑ ... depends on the extent of the plate
☐ ... can be optimized with toothpaste
☐ ... is only achieved through adhesive creams

10. What are characteristics of a unilateral cleft lip and palate?

☑ The nose on the affected side is wider and flatter
☐ The cleft always stops at the alveolar ridge
☐ There is a connection between the mouth and frontal sinus
☑ The cleft formation is asymmetrical

11. What are dental and skeletal consequences associated with cleft lip and palate?

☑ Aplasia in the cleft area
☑ Anterior and lateral crossbite
☐ Buccal non-occlusion
☐ Macrognathie

12. Which three devices are used in the primary treatment of newborns with cleft and Pierre Robin sequence according to the Frankfurt concept?

☐ Functional appliances, passive plates, and Pierre Robin plates
☑ Active plates, passive plates, and Pierre Robin plates
☐ Pierre Robin plates, Functional appliances, and passive plates
☐ Passive plates, self-ligating plates, and Pierre Robin plates

13. At what age are patients with cleft lip and palate treated surgically according to the “Basel / Frankfurt concept”?

☐ Within the first three months of life
☐ Around two years
☐ Only after the mixed dentition phase
☑ Around six months of age

14. What are possible associated symptoms/complications in patients with cleft lip and palate?

☑ Hearing impairments and recurrent otitis media due to eustachian tube dysfunction
☑ Beeinträchtigung der Mimik

☑ Sigmatismus
☐ Astigmatismus

Self-Assessment Questions

Note: Please select how strongly the following statements apply to you; only one answer per item.

I am satisfied with my knowledge about cleft lip and palate.

☐ Strongly agree ☐ Agree ☐ Neutral ☐ Disagree ☐ Strongly disagree

I am interested in the topic of cleft lip and palate.

☐ Strongly agree ☐ Agree ☐ Neutral ☐ Disagree ☐ Strongly disagree

I can name and explain the differences between various types of clefts.

☐ Strongly agree ☐ Agree ☐ Neutral ☐ Disagree ☐ Strongly disagree

I can explain the importance of early intervention and multidisciplinary care in cleft treatment.

☐ Strongly agree ☐ Agree ☐ Neutral ☐ Disagree ☐ Strongly disagree

I have basic knowledge of the role of speech therapists, orthodontists, and other professionals involved in cleft care.

☐ Strongly agree ☐ Agree ☐ Neutral ☐ Disagree ☐ Strongly disagree
